# Supplementary material for: Targeting Prohibitins to Inhibit Melanoma Growth and Overcome Resistance to Targeted Therapies
Source: Cells. 2023 Jul 14;12(14):1855. doi: 10.3390/cells12141855 (PMC10378173; doi:10.3390/cells12141855)
Supplement: Supplementary file 1 [file cells-12-01855-s001.zip › cells-2418165-supplementary.pdf]

Supplementary Tables

Supplementary Table S1. IC50 analyses of four different PHB ligands (MEL 9,41,56 and JI130) in four different melanoma lines with different mutation status.

Cell lines / PHB ligands	HBL<br>KIT mut	MM162<br>WT BRAF/WT NRAS	MM074<br>BRAF mut	MM165<br>NRAS mut
MEL 9	10.6	18.4	7.1	20.2
MEL 41	9.3	13.5	5.6	16.8
**MEL 56**	**6.1**	**8.4**	**5.0**	**13.0**
**JI130**	**0.1**	**0.16**	**0.09**	**0.13**

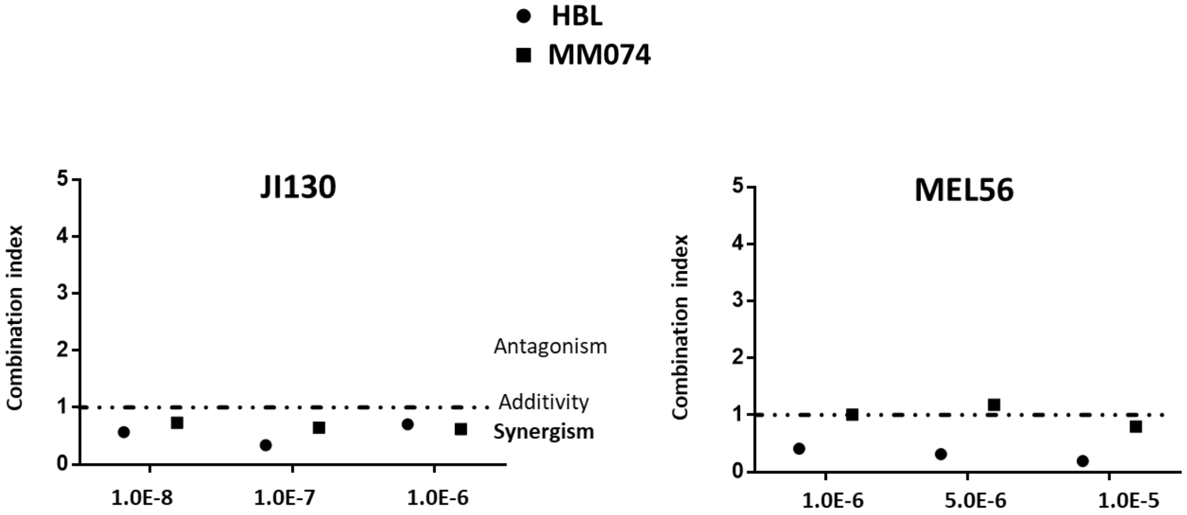

Supplementary Figure S1: Combination of PHB ligands with the autophagy inhibitor chloroquine (CQ). Combination index (CI) plots of the combination of PHB ligands (JI130) and MEL56 with CQ (25 μM) in HBL and MM074 melanoma lines. CI < 1, CI = 1, and CI > 1 indicate synergism, additive effect, and antagonism, respectively.
